# Evaluation of Panoramic Radiography Diagnostic Accuracy in the Assessment of Interdental Alveolar Bone Loss Using CBCT

**DOI:** 10.1002/cre2.70042

**Published:** 2024-11-19

**Authors:** Najmeh Anbiaee, Pedram Pahlavanzadeh

**Affiliations:** ^1^ Oral and Maxillofacial Diseases Research Center Mashhad University of Medical Sciences Mashhad Iran; ^2^ Dentist Private practice Mashhad Iran

**Keywords:** alveolar bone loss, cone‐beam computed tomography, panoramic radiography, periodontal bone loss

## Abstract

**Objectives:**

Alveolar bone loss (ABL) and periodontal lesions are common diseases that have an undeniable effect on teeth maintenance and health. Current diagnostic methods include probing, intraoral radiography, and panoramic radiography; each has its limitations. In this study, we aimed to assess the diagnostic accuracy of panoramic radiography in the diagnosis of interdental ABL.

**Material and Methods:**

In this cross‐sectional study, panoramic and cone‐beam computed tomography (CBCT) images from 80 patients were collected from the archives of an oral and maxillofacial radiology center. The amount of ABL was obtained by measuring the distance from the Cemento‐Enamel Junction (CEJ) to the alveolar bone crest on both panoramic and CBCT images. Patients were divided into healthy (ABL ≤ 2 mm) and diseased (2 mm < ABL) groups in terms of periodontal disease.

**Results:**

There was no statistically significant difference in the average ABL in the premolar, maxillary molar, and mandibular molar areas between the two techniques. However, in other areas, the ABL size was significantly lower in the panoramic view (*p* < 0.05). Also, the panoramic technique correctly recognized 89.1% of normal and 88.4% of ABL cases. The overall accuracy of panoramic radiography in the diagnosis of ABL was 85%, indicating the good accuracy of this technique. In maxilla, the highest diagnostic accuracy of the panoramic technique was in the molars, and the lowest was in the incisors. In the mandible, the highest and lowest diagnostic accuracy of the panoramic technique was related to molars and premolars, respectively. According to the kappa statistic, there was a significant good to very good agreement between the two types of techniques in all maxillary areas (*p* < 0.001).

**Conclusions:**

Panoramic radiography is accurate in showing ABL. Measuring ABL in the posterior mandibular areas in panoramic radiographs is quite reliable; however, in general, digital panoramic radiography shows less ABL than the actual amount.

## 1 Introduction

Alveolar bone loss (ABL) and periodontal lesions are common diseases that have an undeniable effect on teeth maintenance and health and people's quality of life (Page 1998). In addition to oral and dental problems, periodontal diseases can affect a person's general health with symptoms such as coronary artery disease, stroke, diabetes, premature birth, low birth weight, and some respiratory diseases (Hegde and Awan 2019; Mealey 1999).

The normal alveolar crest is approximately between 0.5 and 2 mm below the Cemento‐Enamel Junction (CEJ) surface of adjacent teeth. In posterior teeth, the alveolar crest is parallel to the CEJ of adjacent teeth, whereas in front teeth, the alveolar crest is seen as pointed and sharp. Normally, the connection between the alveolar crest and the lamina dura of the posterior teeth forms a sharp angle adjacent to the root of the tooth (White and Pharoah 2014). Periodontal diseases often manifest initially with a fuzziness or a lack of continuity in the lamina dura in the mesial and distal interdental septa. This condition then progresses to a radiolucent wedge‐shaped area in the interdental septa, and eventually bone destruction presents itself as a decrease in height in this area (Zaki et al. 2015).

When periodontal disease causes bone loss in the branching area of the roots in a tooth with two or more roots, a radiolucent lesion appears in the area of the furcation (Zaki et al. 2015; Scarfe and Farman 2008). Radiographic evaluation in cases where radicular bone is missing can aid in diagnosing the lesion. However, the angle of the irradiated rays may lead to the overlapping of adjacent radiopaque structures, concealing the lesion remains from the clinician's view (Zaki et al. 2015). The depiction of furcation involvement around maxillary molars is not as clear as that of mandibular molars due to the superimposition of the palatal root on the lesion. In the mandible, the external ridge may obscure furcation involvement in third molars. Convergent roots may also mask furcation defects in the second and third molars of both the maxilla and mandible (White and Pharoah 2014).

For diagnosing periodontal diseases, clinical examination and radiology are complementary. To evaluate a patient's periodontal condition, a clinical examination is conducted to determine necessary tests and radiographs. If periodontitis is identified during clinical examination, radiological imaging is recommended. It should be noted that radiographic images may not detect mild bone destruction; typically, significant bone destruction is visible on radiographic images once it has occurred (Zaki et al. 2015; Haas et al. 2018). Panoramic radiography is a common radiograph for examining oral structures. It provides a single image of a wide range of maxillofacial structures, including the nasal cavity and maxillary sinus. This radiography is primarily used for initial screening (Arslan et al. 2020).

In recent years, 3D imaging techniques have gained significant attention. Since its introduction in 1998, cone‐beam computed tomography (CBCT) has become a popular technique in dental diagnosis and treatment processes (Shukla, Chug, and Afrashtehfar 2017). The accuracy of CBCT in measuring bone dimensions surpasses that of panoramic radiography (Sangha et al. 2021). Among radiographic images, CBCT images closely correlate with the direct measurements of CEJ distance from the alveolar bone crest on the skull. Low‐dose CBCT, as a new method of imaging, is a highly accurate and reliable method for detecting and measuring furcation defects by lower radiation dose (Ruetters, Gehrig, Kim, et al. 2022).

In various jaw areas, panoramic radiography magnification varies, with distances measured by panoramic radiography highly correlated with those measured by CBCT (Tang, Liu, and Chen 2017).

There is considerable disparity among researchers regarding the accuracy of panoramic radiography. It has been suggested that the results of the panoramic radiographic evaluation cannot replace clinical evaluation findings, but can serve as a screening tool for periodontal disease (Machado et al. 2020). Also, it has been shown that there is a difference of 0–1.6 mm between clinical and radiographic evaluations in measuring the height of the alveolar crest (Zaki et al. 2015). In contrast, in some studies, no statistically significant difference between panoramic and CBCT was observed (Takeshita et al. 2014).

Correct and accurate diagnosis of periodontal disease can control the disease, preserve the teeth for longer, and improve the quality of life and general health of the person. Current diagnostic approaches such as probing and intraoral and panoramic radiography have limitations due to their errors. The purpose of this study is to investigate the accuracy of panoramic radiography in the diagnosis of ABL and periodontal lesions, which is important considering the high prescription of this type of radiography in patients with periodontal diseases.

## 2 Materials and Methods

### 2.1 Study Design and Ethical Approval

The protocol of this descriptive cross‐sectional study was approved by the Research and Ethics Committee of Mashhad University of Medical Sciences with the approval number IR.MUMS.DENTISTRY.REC.1399.112.

### 2.2 Sample Size Calculation and Data Collection

Panoramic images and CBCT scans of 80 patients, including 42 men and 38 women aged between 18 and 71 years, who were referred by the clinicians for various reasons, were obtained from the archives of a private oral radiology center. Before using the radiographs, we contacted the patients and asked them to sign a written informed consent for using their radiographs for this study. They were assured that their personal information (name, age, and sex) is going to remain private and is not going to be published.

Following Tang, Liu, and Chen's (2017) article, the sample size was determined to be 80 samples using the following formula:

$$n=\frac{{\left(z1-\frac{\alpha }{2}+z1-\beta \right)}^{2}}{c2}+3,$$

$$c=\frac{1}{2}\ln\frac{1+r}{1-r},$$

CBCT images were acquired using a mid‐type CBCT device (Planmeca, Helsinki, Finland) with a field of view (FOV) of 10 × 10 and normal resolution. Panoramic images were obtained using a Planmeca Promax 2DS3 (Planmeca, Helsinki, Finland) device.

### 2.3 Inclusion and Exclusion Criteria

Patients over 18 years old were selected. Only images in which the distance between the CEJ and the alveolar bone crest of existing teeth could be adequately assessed were included as samples. Patients with images of insufficient quality or clarity due to implants or metal objects or those with cysts or tumors that hindered examination were excluded. Completely edentulous patients and those with systemic bone‐related diseases were also excluded.

The samples were evaluated for the health status of the alveolar bone crest, the presence of ABL, and furcation involvement in existing teeth, using both panoramic radiography and CBCT techniques. The amount of bone loss (distance between CEJ and alveolar bone crest) was measured in both types of radiographs using Planmeca Romexis viewer version 3.8.0.R software, labeled as ABL in the study.

### 2.4 Assessment of ABL on the Basis of Radiographs

**Figure 1:**
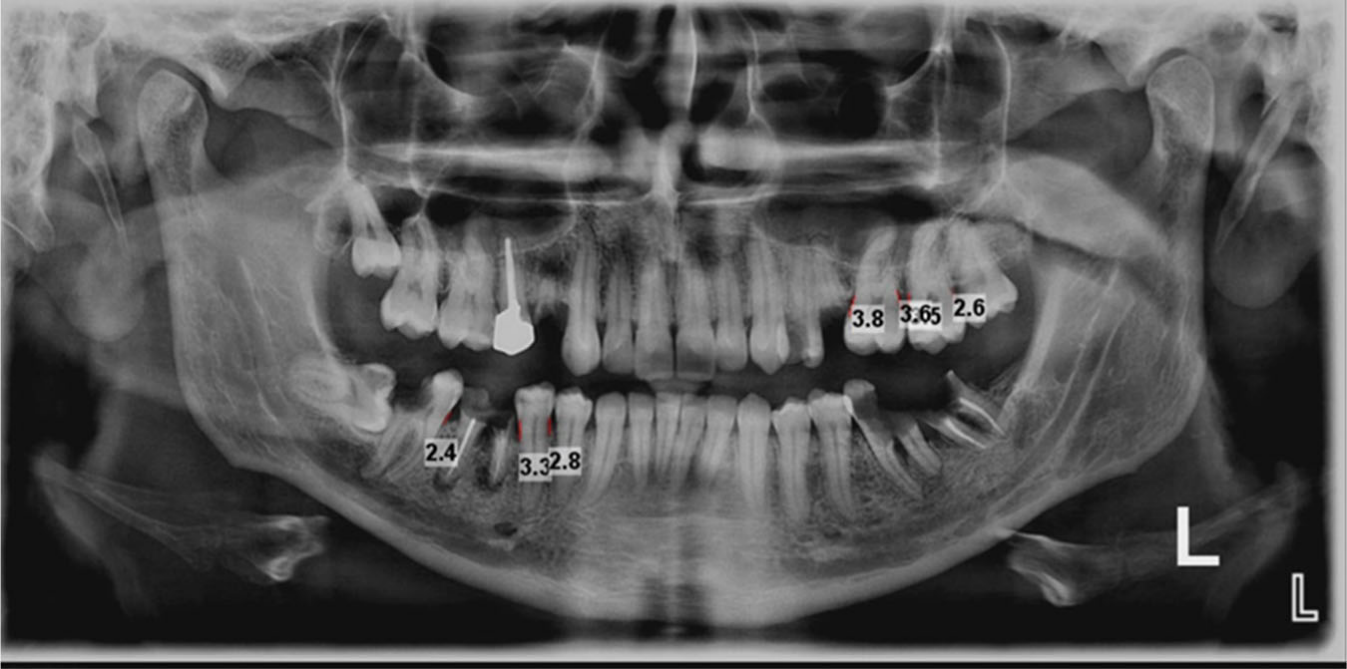
Measuring bone loss in panoramic images.

Initially, a trained operator examined the patient's panoramic images in real size without resizing using Planmeca Romexis viewer version 3.8.0.R software (Figure 1). The operator could adjust the image contrast for better visualization of the alveolar bone crest. The distance between the CEJ of the teeth and the alveolar bone crest was measured to determine bone resorption. In cases where the CEJ was not clearly visible, the distance from the repair margin to the alveolar bone crest was considered. A subset of 10% of samples was re‐evaluated by the operator after 2 weeks.

**Figure 2:**
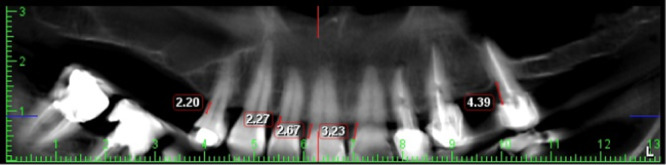
Measuring bone loss in panoramic view of CBCT images.

Because buccal and lingual tooth surfaces are not captured in panoramic radiographs, only bone loss on proximal tooth surfaces was assessed in panoramic images. To illustrate this view, a panoramic‐like view of CBCT images was drawn separately for the upper and lower jaw (Figure 2). The same surfaces were evaluated in CBCT scans to allow for accurate comparison and measurement of panoramic accuracy. Measurements were taken parallel to the long axis of the teeth at proximal levels in the CBCT images, and ABL was measured from the CEJ to the alveolar bone crest in the same view (Takeshita et al. 2014).

### 2.5 Statistical Analysis

Data from CBCT images were recorded in SPSS version 26.0 software. Samples were categorized into healthy (ABL ≤ 2 mm) and diseased (2 mm < ABL) groups based on the amount of bone loss. Data were recorded and analyzed using SPSS, along with Shapiro–Wilk, Wilcoxon, and Kappa statistical tests.

The Shapiro–Wilk test indicated that data distribution was non‐normal across all areas (*p* < 0.05).

## 3 Results

In this research, simultaneous panoramic and CBCT images of 80 patients, including 38 women (47.5%) and 42 men (52.5%) with a mean age and standard deviation of 47.3 ± 13.5 years and an age range of 18–71 years, were evaluated for bone crest status. The presence and amount of ABL in the mesial and distal surfaces of the patient's existing teeth, as well as furcation involvement, were also evaluated. A total of 1646 dental proximal surfaces were assessed and measured using both panoramic and CBCT techniques.

Intraexaminer agreement was deemed acceptable and there was no statistically significant difference in the re‐measurement of samples by the operator (*p* > 0.05).

There was no statistically significant difference in the average ABL between the two techniques in the premolar and molar areas of the maxilla and molar areas of the mandible. However, in other areas, the ABL size was significantly higher in CBCT (*p* < 0.05; Table 1).

**Table 1:** Comparison of two types of imaging techniques in diagnosing patients (2 mm < ABL) by area and jaw.

Jaw	Area	Type of image	Number	Mean ± standard deviation of ABL^b^	(Highest/lowest) median ABL	*p* value
Upper	Incisor	CBCT^a^	43	2.7 ± 0.5	2.7 (2.1, 4.1)	0.013
		Panoramic	48	2.5 ± 0.6	2.5 (1.9, 4.2)	
	Canine	CBCT	18	3.0 ± 1.2	2.7 (2.2, 8.0)	0.016
		Panoramic	13	2.3 ± 0.5	2.2 (1.9, 3.8)	
	Premolar	CBCT	33	3.0 ± 0.8	2.9 (2.1, 5.7)	0.082
		Panoramic	35	2.6 ± 0.7	2.4 (1.9, 4.1)	
	Molar	CBCT	60	2.8 ± 0.8	2.5 (2.1, 5.4)	0.470
		Panoramic	57	2.9 ± 0.9	2.7 (1.9, 6.0)	
Lower	Incisor	CBCT	147	4.3 ± 2.3	3.7 (2.1, 13.2)	< 0.001
		Panoramic	113	3.9 ± 2.3	3.1 (1.9, 11.1)	
	Canine	CBCT	61	4.0 ± 2.2	3.1 (2.1, 12.7)	0.011
		Panoramic	53	3.7 ± 1.9	3.0 (1.9, 10.0)	
	Premolar	CBCT	51	3.3 ± 1.5	2.7 (2.1, 7.5)	0.003
		Panoramic	48	3.1 ± 1.4	2.6 (1.9, 7.3)	
	Molar	CBCT	21	3.2 ± 1.0	3.0 (2.1, 5.8)	0.452
		Panoramic	20	3.0 ± 0.9	2.9 (1.9, 5.9)	

^a^ Cone‐beam computed tomography.

^b^ Alveolar bone loss.

Table 2 displays healthy individuals (ABL ≤ 2 mm) and patients (2 mm < ABL) based on the type of radiography and the results of diagnostic indices, including sensitivity, specificity, accuracy, and overall agreement value by area and jaw. In the maxilla, the highest and lowest diagnostic accuracy of the panoramic technique was observed in the molar area and the incisor area, respectively. According to the Kappa statistic, there was very good agreement in the molar area and good to fairly good agreement in other areas between the two types of radiographic techniques. In the mandible, the highest and lowest diagnostic accuracy of the panoramic technique was related to molar and premolars, respectively. According to the Kappa statistic, there was good agreement between the two types of radiographic techniques in all areas. All in all, regardless of the jaw, the highest accuracy was related to maxillary molars, and the lowest was related to maxillary incisors. Significant agreement was observed in all areas and both jaws based on the Kappa statistic (*p* < 0.01 for each).

**Table 2:** Bone crest condition and diagnostic indicators.

Jaw	Area	CBCT^a^		Panoramic		Sensitivity	Specificity	Accuracy	Kappa	Agreement
		Healthy	Unhealthy	Healthy	Unhealthy					
Maxilla	Incisor	229	43	224	48	0.638	0.945	0.792	0.609	Good
	Canine	93	18	98	13	0.769	0.917	0.834	0.588	Relatively Good
	premolar	186	33	194	25	0.875	0.941	0.908	0.713	Good
	Molar	153	60	156	57	0.893	0.941	0.917	0.820	Very Good
Mandible	Incisor	167	147	199	113	0.982	0.824	0.903	0.760	Good
	Canine	92	61	100	53	0.923	0.878	0.901	0.673	Good
	Premolar	189	51	194	48	0.851	0.937	0.894	0.657	Good
	Molar	103	21	104	20	0.850	0.961	0.906	0.795	Good

^a^ Cone‐beam computed tomography.

The overall agreement value of the two types of radiographs based on the Kappa statistic was 0.73, indicating relatively good agreement. The overall accuracy of panoramic radiography in diagnosing bone loss was calculated to be 85%, suggesting high accuracy in diagnosing bone loss.

Regarding furcation involvement, 109 molar teeth in the maxilla of the patients were examined using both CBCT and panoramic images. Among these, five teeth were diagnosed with furcation involvement in CBCT images, whereas panoramic images detected furcation involvement in four cases (80%) of these lesions. In the lower jaw, 73 molar teeth were examined using both techniques, in CBCT images, and eight teeth were diagnosed with furcation involvement, with panoramic images detecting seven cases (87.5%) of them.

## 4 Discussion

Due to the low cost, availability, and minimal patient dose, digital panoramic radiography is widely used as a valuable tool in diagnosing periodontal disease, despite its limitations. Disagreement regarding the accuracy of this radiograph persists, underscoring the importance of determining its precision in evaluating periodontal lesions.

Babaloo et al. (2015) demonstrated no difference between CBCT imaging and clinical examination. Hass et al. also reported in 2017 that the measurements obtained from CBCT were comparable to direct measurements, establishing CBCT as the gold standard due to its high accuracy in detecting vertical and horizontal measurements, as well as bone loss (Haas et al. 2018). Ruetters et al. showed that low‐dose CBCT seems to be a precise method for describing buccal bone and its thickness adjacent to mandibular anterior teeth in this experimental setting (Ruetters, Gehrig, Kronsteiner, et al. 2022).

In this study, only bone loss on the proximal surfaces of the teeth was examined in panoramic and CBCT images for comparative accuracy assessment, as panoramic radiographs do not show the buccal and lingual surfaces of the teeth.

The results of the present study showed that although there is good and very good agreement between panoramic radiography and CBCT in detecting the amount of bone loss, the average amount of bone loss in panoramic radiography was lower than in CBCT. This difference may be due to several reasons. Despite considering the beam geometry, the panoramic radiography device has a magnification of 1.22–1.3, which is lost in digital radiography images due to calibration of the device and isometricizing the pixels of the image receiver sensor (Hermann et al. 2019; Moreira‐Souza et al. 2022; Razi, Moslemzade, and Razi 2009).

Several studies have investigated the magnification of digital and conventional radiography images. Razi et al. who examined CC Planmeca and Panoura devices concluded that both conventional radiography devices have a magnification of about 1.1. As seen in conventional radiography techniques, the images have inherent magnification. In contrast, Abdinian et al. (2016) found negative magnification (−6.4% for Planmeca Promax Scara3 and −2.6% for Instrumentarium P200) in digital panoramic radiographs. In both devices, the average vertical dimensions in the obtained images were lower than the actual size in all facial areas. In our study, which was performed using the Scara3 panoramic radiography device, it seems that the images have negative magnification.

The difference in magnification among different radiography devices may be due to variations in the shape, number, and size of the focal troughs and rotation mechanism (Abdinian et al. 2016). Additionally, the type of image processing software in different digital panoramic radiography devices can influence magnification.

Another reason for the lower bone height loss in panoramic images compared to CBCT could be the overlapping of anatomical areas in panoramic images, which is eliminated in CBCT. If the alveolar bone crest level in the buccal or lingual area is higher than the maximum bone loss between two teeth, the overlapping can result in an underestimation of bone loss in panoramic radiographs (Kim et al. 2011).

In panoramic radiography, the ray is exposed with an angle of −7 degrees upward. This negative angle can also be effective in imaging the alveolar bone crest and showing less bone loss. In some studies, even with the use of conventional panoramic radiographs, which have inherent magnification, the amount of bone loss is lower than its actual amount. In Takeshita's study, which examined the accuracy of various radiographs, including panoramic radiographs, the average amount of bone loss in conventional panoramic radiographs (2.66 mm) was found to be less than the actual amount (3.01 mm).

In the present study, the lowest level of accuracy in panoramic radiography for diagnosing ABL was observed in the maxillary incisor area, whereas the highest accuracy was noted in the maxillary molar region. No similar studies were identified that specifically investigated the accuracy of panoramic radiography in different jawbone areas as comprehensively as our study.

Statistical analysis revealed no significant difference between panoramic radiography and CBCT in detecting ABL in the posterior maxilla (premolar and molar regions) and the posterior mandible (molar region). This finding suggests that panoramic radiography is highly reliable in accurately identifying the extent of bone loss in these specific areas. Overall, there was good to very good agreement across all jaw regions between panoramic images and CBCT scans, supporting the use of panoramic radiography as a valuable tool for assessing ABL.

In our investigation, panoramic radiography successfully identified furcation involvement in 80% of cases in the upper jaw and 5.87% in the lower jaw. These detection rates are consistent with those reported by Komšić et al. (2019), who found a furcation involvement detection rate of 63%.

## 5 Conclusion

The results of the study mentioned earlier demonstrate that panoramic radiography is accurate in depicting periodontal disease or periodontal tissue health. In the upper jaw, the OPG technique exhibits the highest diagnostic accuracy in the molar areas and the lowest in the premolar areas. While measuring the amount of bone loss in the posterior maxillary regions with panoramic radiographs is entirely reliable; overall, panoramic radiography tends to underestimate the actual extent of bone loss.

## Author Contributions

N.A. was responsible for the conceptualization and supervision. P.P. was responsible for assessments. N.A. conducted the statistical analysis. N.A. and P.P. wrote and edited the manuscript. All authors reviewed and approved the final manuscript.

## Conflicts of Interest

The authors declare no conflicts of interest.

## Data Availability

The data of the current study are available from the corresponding author upon request.
